# Measuring Prosocial Behaviors: Psychometric Properties and Cross-National Validation of the Prosociality Scale in Five Countries

**DOI:** 10.3389/fpsyg.2021.693174

**Published:** 2021-07-22

**Authors:** Bernadette Paula Luengo Kanacri, Nancy Eisenberg, Carlo Tramontano, Antonio Zuffiano, Maria Giovanna Caprara, Evangelina Regner, Liqi Zhu, Concetta Pastorelli, Gian Vittorio Caprara

**Affiliations:** ^1^Department of Psychology, Pontificia Universidad Católica de Chile, Santiago, Chile; ^2^Department of Psychology, Arizona State University, Arizona, AZ, United States; ^3^Centre for Research in Psychology, Behavior and Achievement, Coventry University, Coventry, United Kingdom; ^4^Department of Psychology, Sapienza Università di Roma, Rome, Italy; ^5^Department of Psychology and Health, Open University of Madrid, Madrid, Spain; ^6^Universidad Católica de Santa Fe, Santa Fe, Argentina; ^7^Institute of Psychology, Chinese Academy of Sciences, Guangzhou, China

**Keywords:** cross-cultural assessment, psychological assessment, bi-factor model, prosociality, prosocial behavior, empathy, helping behaviors

## Abstract

This research investigated the psychometric properties of the Prosociality Scale and its cross-cultural validation and generalizability across five different western and non-western countries (China, Chile, Italy, Spain, and the United States). The scale was designed to measure individual differences in a global tendency to behave in prosocial ways during late adolescence and adulthood. Study 1 was designed to identify the best factorial structure of the Prosociality Scale and Study 2 tested the model’s equivalence across five countries (*N* = 1,630 young adults coming from China, Chile, Italy, Spain and the United States; general *M*_age_ = 21.34; *SD* = 3.34). Findings supported a bifactor model in which prosocial responding was characterized by a general latent factor (i.e., prosociality) and two other specific factors (prosocial actions and prosocial feelings). New evidence of construct validity of the Prosociality Scale was provided.

## Introduction

Given the current mass migrations of people, often resulting in social exclusion and conflict, it is important to identify human behaviors that can foster greater cohesion among different groups in increasingly multicultural societies. Prosocial behaviors (i.e., voluntary, desirable actions aimed at benefit others such as sharing, consoling, and helping; see Penner et al., 2005; Eisenberg et al., 2015) may reduce prejudice, improve attitudes towards others, and produce positive and inclusive social interactions (e.g., Yates and Youniss, 1996; Batson, 2011; see Eisenberg et al., 2010). Empirical research has also provided evidence that prosocial behavior predicts individuals’ well-being, personal adjustment, and successful youth development (e.g., Lerner et al., 2005), perhaps because it counteracts and protects children from experiences likely to elicit depression and conduct problems (e.g., Bandura et al., 1999) and is related to scholastic achievement (e.g., Wentzel, 1993; Caprara et al., 2000, 2015).

Despite its societal and individual relevance, how to operationalize and assess prosocial behavior is still a matter of debate (see Eisenberg et al., 2015). At present, reliable measures for studying dispositional differences in prosocial behavior in late adolescence and adulthood are relatively scarce. Moreover, given the diversity of cultural groups in many societies, it is useful to develop instruments applicable for multicultural contexts and to consider issues related to measuring constructs across cultural groups. To this end, we conducted two studies designed to further examine the psychometric properties of the Prosociality Scale, and especially its generalizability across different western and non-western countries.

This scale was originally developed in Italy (Caprara and Pastorelli, 1993; Pastorelli et al., 1997) and was designed to measure the general and global tendency to react in prosocial ways during late adolescence and adulthood. Study 1 was designed to identify the best factorial structure of the Prosociality Scale by testing four different alternative models in an Italian sample of young adults. Study 2 tested the psychometric properties of the best model in Study 1 and the equivalence of the model across China, Chile, Spain, and the United States. In addition, using the Italian and Chinese samples, additional evidence of the convergent validity of the Prosociality Scale was provided.

## The Assessment of Prosocial Behaviors

In recent decades, scholars have advocated a range of methods to assess prosocial behaviors (Tremblay et al., 1992; Penner and Finkelstein, 1998; Carlo and Randall, 2002; Roche Olivar, 2002; Inglés et al., 2003). It is beyond the scope of the present paper to discuss the benefits and limits of all different plausible measures. However, questionnaires, because they are more feasible to administer and score than most behavioral/experimental measures of prosocial behavior, offer the possibility of assessing prosocial behaviors in large samples, including large intervention projects, and can be relatively easily tested for their generalizability across cultures. Compared to the body of research conducted with children, the study of age-related change in prosocial behavior from adolescence to adulthood, and predictors and sequelae of such behavior, is limited (see Eisenberg et al., 2009). As a consequence, information regarding the prediction of prosocial behavior and beneficial outcomes of behaving prosocially in emerging and early adulthood is still scarce. Whereas the assessment of prosocial behavior in children often has involved observational data or reports of prosocial behavior and emotion by multiple informants (e.g., Vaish et al., 2009), measures of adults’ prosocial behaviors have principally been self-reported (e.g., Carlo and Randall, 2002). Scholars who support the value of using self-reports for the assessment of adults’ prosocial behaviors have claimed that, given the socio-cognitive development that occurs in adolescence, no one can report as accurately on people’s habits and tendencies to behave prosocially as the individuals themselves (Caprara et al., 2012).

Existing measures of prosocial behavior have often assessed specific dimensions of prosocial behavior such as helping (e.g., Bar-Tal, 1982; Beauchaine et al., 2013), donating or sharing (e.g., Stewart and McBride-Chang, 2000; French et al., 2011), or empathic concern/feelings (e.g., Davis and Franzoi, 1991; Batanova and Loukas, 2012). However, investigators often want to assess a broad range of prosocial behaviors. Assessment of a specific type of prosocial behavior is particularly useful when identifying psychological mechanisms, motivations, and contextual processes involved in that particular kind of behavioral responding. In contrast, assessment of the broad propensity to act in favor of others (i.e., prosociality) is likely to tap a disposition that is less dependent on specific needs, situations, or reactions to specific others’ needs. Underlying the latter assessment approach is the idea that individuals have a certain inclination to act (or not to act) in a manner that alleviates others’ distress and that these individual differences account for a portion of variability in prosocial behaviors and in its stability across time.

Longitudinal research following children from early childhood to adulthood supports the existence of the long-debated altruistic or prosocial personality (e.g., Eisenberg et al., 2014). Today researchers converge in considering prosocial behavior as affected indirectly by individual differences in biological (e.g., Knafo and Plomin, 2006), sociocognitive (e.g., Vaish and Warneken, 2012), and temperamental features (e.g., McGinley, 2008), differential socialization experiences (i.e., in the family, school, and community; see Eisenberg et al., 2015), and specific characteristics of a given situation (e.g., who is the recipient of the prosocial action, Carlo et al., 2003). These “ingredients” all appear to have an effect on how people perceive and interpret specific other’s needs or contingencies and on how they regulate their responding and decide to act when they have opportunities to engage in prosocial actions.

In summary, self-report measures of prosocial behavior can be classified into those that assess global prosocial behavior or those that assess prosocial behavior in specific situations or contexts (e.g., Carlo et al., 2003). Most of the existing available measures to assess prosocial behavior globally include prosocial behavior as a component/factor of a broad questionnaire assessing other relevant adjustment/maladjustment dimensions of children’s or adolescents’ development. In general, these scales were created for clinical purposes (e.g., Taylor and Wood, 2014). For example, the frequently used and cross-nationally validated *Strengths and Difficulties Questionnaire* (SDQ; Goodman, 1999; Van Roy et al., 2008) assesses prosocial behavior with a 5-item subscale. Psychometric evaluations of this questionnaire have found satisfactory convergent and discriminant validity, whereas factor analytic studies have obtained mixed results across countries regarding the five factors hypothesized [i.e., four factors related with difficulties: hyperactivity, emotional symptoms, conduct problems, peer problems; and one related with strengths: prosocial behavior (Goodman et al., 2003)]. More recently, the SDQ was also evaluated in a large community sample of Norwegian pre-, early, and late adolescents (ranging in age from 10 to 19) and a positive construal factor (i.e., self-reported prosocial behaviors) was identified, but had a modest effect compared with the other four traits (Van Roy et al., 2008). These results suggested to these authors that the meaning of the prosocial subscale was unclear and that there was a need to improve its internal reliability and conceptual clarity (Van Roy et al., 2008).

In addition, self-reported measures of prosocial tendencies and behaviors for general samples have sometimes been included in the assessment of general social skills, such as the *Values in Action Inventory of Strengths* (*VIA-IS*; Peterson et al., 2005) that considers a subscale of the interpersonal strengths of “tending and befriending” others. However, these scales often assess only narrow domains of prosocial behavior. To our knowledge, the Penner and Finkelstein’s (1998) scale is one of the only existing instruments in which there is a specific focus on prosocial responding such as helping behaviors and empathy-related dimensions. Their scale, called *Prosocial Personality Battery* (PSB), consists of 56 items divided into seven individual subscales (i.e., social responsibility, empathic concern, perspective taking, personal distress, other-oriented moral reasoning, mutual moral reasoning, and self-reported altruism). These subscales were psychometrically analyzed by Penner et al. (1995), resulting in the seven subscales loading onto two factors (other-oriented empathy mean tendency and helpfulness mean tendency). The PSB is a commonly used scale in social psychology for assessing prosocial tendencies from a personality trait perspective. However, to our knowledge, the instrument was analyzed psychometrically and validated only in the US samples of adults (Penner et al., 1995), college students (Ruci, 2011), and a clinical sample (Pagano et al., 2010).

In summary, reliable and universally applicable measures for the evaluation of the global tendency to perform prosocial behavior are needed, especially considering the vacuum in the assessment of prosociality from late adolescence to adulthood. For this reason, we sought to provide cross-cultural evidence regarding the psychometric properties of an existing scale for assessing the broad domain of prosocial behavior for use with older adolescents as well as adults (i.e., the Prosociality Scale).

## The Prosociality Scale

In Italy, based on a measure to assess prosocial tendencies in children (Caprara and Pastorelli, 1993). Caprara et al. (2005a) developed a 16-item scale to assess the global propensity to behave prosocially from late adolescence to adulthood. In this revised scale, items were reworded to be adequate for adolescents and adults and new items related to empathic reactions were added. Although not all prosocial behavior involves empathy/sympathy, the authors argued that including the empathic feelings dimension is necessary because “…in adulthood, one’s empathic motives or predispositions are not merely a correlate of his or her tendency to act prosocially but, rather, an integral part of such a tendency” (Caprara et al., 2005b, p. 80).

The Prosociality Scale was designed as a measure to assess individual differences in general adults’ tendencies to act in favor of others and has been proved useful in several studies in different countries (e.g., Bandura et al., 1999; Cuadrado et al., 2015; Pastorelli et al., 2015; Martí-Vilar et al., 2020). The scale has been validated in Italy with classical test theory (Caprara et al., 2005a) and the item response theory approach (Caprara et al., 2005b), showing adequate psychometric qualities and construct validity. In general, the Prosociality Scale has been correlated with agreeableness and emotional and empathic self-efficacy (e.g., Alessandri et al., 2009). A recent study evaluates the psychometric functioning of the Prosociality Scale in three Spanish-speaking countries: Argentina, Spain, and Peru, focusing on university participants (Martí-Vilar et al., 2020). However, the psychometric properties of the Prosociality Scale for late adolescents and adults have not yet been investigated cross-nationally by comparing data from different western and non-western cultures; nor has the scale been tested by considering different alternative models for an understanding of its best factorial structure. Indeed, in a previous psychometric study (Caprara et al., 2005a), exploratory factorial analyses was performed for Italian adults and a one-factor solution was proposed. In that work, the authors used one of the most common and traditional ways to assess dimensionality, that is, to compare the percentages of variance explained by the first and the second unrotated components in a principal component analysis. In that case, the ratio was about 5:1, supporting the unidimensionality of the scale (Caprara et al., 2015).

What has not yet been tested at a confirmatory level is whether the multidimensionality of the scale is reflected in its psychometric structure. As posited by Caprara et al. (2005a), it is plausible to hypothesize that several kinds of prosocial actions (i.e., helping, caring, and sharing) represent a general behavioral dimension, distinct from an affective dimension (i.e., empathic feelings) that often motivates other-oriented prosocial behavior. These two dimensions may operate in concert and influence each other, while also being subsumed by a superordinate factor reflecting the general tendency to be oriented to the needs of others.

## Prosocial Behavior: Testing for Cross-National Invariance

The influence of culture on prosocial tendencies is undoubtedly complex and many scholars have tried to clarify how the broader social environment and specific cultural contexts shape the tendency to interact in prosocial ways (e.g., Fiske, 2004; Batson, 2011). For example, in some more collectivistic cultures, prosocial tendencies are fostered and promoted (Graves and Graves, 1983; Eisenberg and Mussen, 1989), whereas in others, hostility is the norm and prosocial behaviors are unusual (Rohner, 1975). Cross-cultural studies have shown significant variation in prosocial actions (Caprara et al., 2001), particularly in sharing or donating behaviors (Carlo et al., 2001; Pilgrim and Rueda-Riedle, 2002). The lack of instruments validated cross-nationally has often proved an obstacle to examining differences in prosocial tendencies across diverse cultural settings, as well as in delineating commonalities and differences for subgroups within cultural contexts. Of importance, one of the main priorities in cross-national studies is to verify the equivalence of the scales used in order to test whether culturally specific factors exert any influence on the measurement process (Hui and Triandis, 1985).

Building upon previous results in the validation study of the Prosociality Scale (Caprara et al., 2005a,b), the present studies sought (1) to expand analysis of the psychometric properties of the scale by testing the factorial structure using confirmatory factor analysis (CFA) for comparisons among competing models in the context where the scale was developed; (2) to test the generalizability of the latent factorial structure to five different countries; and (3) to examine the construct validity of the scale in two countries characterized by a very different cultural, social, and political environments (Italy and China).

## Study 1

**Figure 1:**
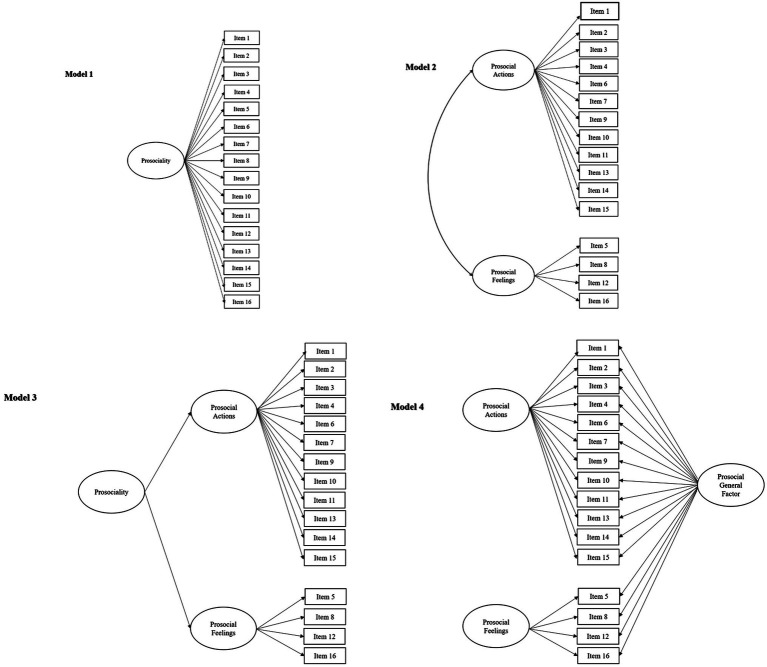
Plausible factorial models for the Prosociality Scale.

The primary aim of this study was to ascertain the best-fitting factorial structure of the Prosociality Scale in an Italian sample (where it was initially developed) to serve as the basis to test its generalizability to non-Italian samples. In particular, as depicted in Figure 1, four alternative models discussed in the literature were compared. Model 1 was the one-factor model, which was the expectation of the authors who developed the scale and was found to be adequate in a prior exploratory analysis (Caprara et al., 2005a). The one-factor model reflects the conceptual argument that prosociality in any form represents a global tendency to behave in favor of others and to alleviate others’ concerns or needs. Model 2, 3 and 4 tested the multidimensional nature of the scale. Originally, Caprara et al. (2005b) addressed prosociality under the assumption that its different facets could be traced to a unique dimension. Nonetheless, those authors also stated that “(…) the generating criteria for this instrument relied heavily on the recommendations of existing developmental literature, which have clearly indicated how prosocialness primarily finds expression in actions of helping, sharing, taking care of, and feeling empathic with others” (Caprara et al., 2005b, p. 78). However, the multidimentionality of the scale has not been tested. Thus, Model 2 included two correlated factors in which different kinds of prosocial actions (i.e., helping, caring, sharing) represent a general behavioral dimension that is associated, even if different than the more affective empathic dimension (i.e., empathic feelings). The existence of these two factors, henceforth labeled PA (prosocial actions) and PF (prosocial feelings), reflects the difference between behavioral tendencies and empathic responses. In the two following models, these two related domains (PA and PF) were hypothesized to be associated to a general construct, which represents the broad tendency to act in favor of others. Model 3 assessed the plausibility of a hierarchy among two specific factors and a general construct, that is, a second-order factor model in which the first level is composed by the two factors (PA and PF) and the second level is a latent factor that captured their contributions to a common general dimension representing the tendency to act prosocially.^1^ Finally, in Model 4, a bifactor approach was used (Chen et al., 2006; Reise et al., 2007), including two domain-specific factors (PA and PF) and a third factor reflecting the communality of all items [i.e., a general prosocial factor (GPF)]. In bifactor models, the various factors are uncorrelated and each item is explained by the appropriate specific factor, plus the general factor that captures individuals’ broad (i.e., general) disposition. Researchers have provided arguments of how a second-order model differs from a bifactor model (e.g., Chen et al., 2006; Reise et al., 2007). In bifactor models, a general factor is hypothesized to account for the commonality of the items, and, at the same time, there are other domain-specific factors, each of which, over and above the general factor, account for the unique role of the specific domain. Instead, in second-order models, the lower-order factors are considerably correlated with each other, and there is a higher-order factor that is hypothesized to account for the relations among the lower-order factors (Chen et al., 2006). In sum, in a second-order factor there is a qualitatively different type of dimension (i.e., a super-ordinate dimension), whereas in a bifactor model, the general factor is on the same conceptual level as the specific factors and represents another possible source of item variance. Because, to our knowledge, no prior studies have tested the way by which a general prosocial tendency is related with specific empathic feelings and prosocial actions, we tested both models (i.e., the second-order model and the bifactorial model) in order to shed light on the plausible multidimensional structure of the Prosociality Scale.

Finally, the secondary aim of this study was to generate evidence regarding the construct validity of the Prosociality Scale. Anchored to theoretical reasons and based on previous findings (see Eisenberg et al., 2015), in order to provide evidence of convergent validity, we computed correlations of the GPF and specific factors (PA and PF) with agreeableness (e.g., Graziano and Tobin, 2002; Luengo Kanacri et al., 2014a), empathy-sympathy (Eisenberg et al., 2010), personal values of benevolence and universalism (Caprara et al., 2012), the quality of friendships (Zuffianò et al., 2014), and aggression (e.g., Kokko et al., 2006). Based on prior evidence and face validity, we expected moderate-to-high positive correlations of the Prosociality Scale with agreeableness, empathic concern and perspective talking dimensions of empathy, self-transcendence values (benevolence and universalism), and the quality of friendships, as well as a moderate-to-high negative correlation between the Prosociality Scale and aggressive behavior.

### Method

#### Participants

The Italian participants were 358 young adults (39% males), ranging in age from 23 to 33 years (*M*_age_ = 25.50; *SD* = 1.64) involved in an ongoing longitudinal study at Genzano, a residential community near Rome (although the data used in this study were cross-sectional). According to national statistics, our sample reflected Italian society in terms of the sociodemographic and occupational characterization of the population (Istituto Italiano di Statistica, 2012). In particular, youths’ employment status of this sample was similar to that in other Mediterranean countries, in which the 47.3% were employed, the 44.9% attended the university, and the 7.7% attended university and were working at the same time.

#### Procedures

Participants from the Italian sample were contacted by phone and invited to participate in the study, for which they received a small payment (the equivalence of a meal). Questionnaires were sent to participants by mail. Questionnaires and consent forms were returned by participants to the researchers during specifically scheduled meetings in a school of Genzano. The study received the approval of the research ethics board of the leading University of (*blinded for review*).

#### Measures

##### Prosociality Scale

Participants rated (1 = *never/almost never true*; 2 = *occasionally true*; 3 = *sometimes true*; 4 = *often true*; 5 = *almost always/always true*) their tendencies to enact prosocial behaviors on the 16-item scale developed by Caprara et al. (2005a). The scale (see content of the items in Table 1) was developed in Italy and reflects different types of prosocial behavior (i.e., sharing, helping, and caring behaviors), as well as empathic/sympathetic reactions. The psychometric properties of the Prosociality Scale have been validated in Italian samples of adults (Caprara et al., 2005a,b). Prior studies have supported the construct validity of the scale, showing theoretically expected correlations of prosocial scores with agreeableness, emotional, and empathic self-efficacy (e.g., Alessandri et al., 2009), self-esteem (e.g., Zuffianò et al., 2014), and civic engagement (e.g., Luengo Kanacri et al., 2014b). Researchers have also found a moderately high correlation (*r* = 0.50) between self- and other-report ratings on this prosociality measure, further supporting its validity (Zuffianò et al., 2014). In this sample, Cronbach’s alpha for the entire scale was 0.94.

**Table 1:** Standardized factor loadings in the bifactorial model from confirmatory factor analysis (CFA) of the Prosociality Scale in the Italian sample (Study 1).

Items of the Prosociality Scale	GPF	SF
PA		
1) I am pleased to help my friends/colleagues in their activities	0.649^*^	0.289^*^
2) I share the things that I have with my friends	0.634^*^	0.411^*^
3) I try to help others	0.740^*^	0.162^*^
4) I am available for volunteer activities to help those who are in need	0.550^*^	−0.101
6) I help immediately those who are in need	0.820^*^	−0.046
7) I do what I can to help others avoid getting into trouble	0.799^*^	−0.065
9) I am willing to make my knowledge and abilities available to others	0.746^*^	0.154^*^
10) I try to console those who are sad	0.790^*^	0.066
11) I easily lend money or other things	0.603^*^	0.378^*^
13) I try to be close to and take care of those who are in need	0.860^*^	−0.020
14) I easily share with friends any good opportunity that comes to me	0.656^*^	0.541^*^
15) I spend time with those friends who feel lonely	0.694^*^	0.395^*^
PF		
5) I am emphatic with those who are in need	0.724^*^	0.555^*^
8) I intensely feel what others feel	0.701^*^	0.374^*^
12) I easily put myself in the shoes of those who are in discomfort	0.708^*^	0.351^*^
16) I immediately sense my friends’ discomfort even when it is not directly communicated to me	0.612^*^	0.151^*^

SF, Specific Factors; PA, Prosocial Actions; PF, Prosocial Feelings; GPF, General Prosocial Factor.

* *p* ≤ 0.05.

##### Other Measures

In order to assess convergent validity, self-reports regarding relevant variables were used. Agreeableness was evaluated *via* the 13-item domain subscale of the short version of the Big Five Questionnaire (Caprara et al., 1993; *α* = 0.71). Six items of The Friendship Qualities Scale (Bukowski et al., 1994) were used to assess the quality of friendship (*α* = 0.88). The self-transcendence values were evaluated using the 4-items subscales (i.e., benevolence and universalism) of the Portrait Values Questionnaire (Schwartz et al., 2001; *α*_s_ = 0.91 and 94, respectively). Moreover, two dimensions of empathy were assessed through Interpersonal Reactivity Index (Davis, 1983) subscales of empathic concern and perspective talking (7-items each; *α*_s_ = 0.76 and 0.81, respectively). Finally, aggression was measured using the 18-item subscale of the Youth Self Report (Achenbach, 1991; *α* = 0.82). The scores for all these variables were computed as the mean of each scale’s items (reversing items as appropriate).

#### Analytical Approach

To test the four competing models described above, confirmatory factor analyses were performed using Mplus 7.11 (Muthén and Muthén, 2012). The maximum likelihood estimator for continuous variables was employed. Evaluation of goodness of fit of the models was based on indices that are less sensitive to sample size (Browne and Cudeck, 1993; Kline, 2005): (1) the root-mean-square error of approximation ranging from 0 to 1 (<0.05 indicates good fit; <0.08 indicates acceptable fit; Browne and Cudeck, 1993) with associated 95% confidence intervals (CIs); (2) the comparative fit index (CFI) ranging from 0 to 1 (>0.90 indicates acceptable fit; >0.95 indicates good fit; Bentler and Bonett, 1980); and (3) and the Tucker–Lewis Index ranging from 0 to 1 (>0.95 indicates good fit; >0.90 indicates acceptable fit; Bentler and Bonett, 1980). In addition, to compare the alternative nonnested models proposed, we considered the Akaike information criterion (AIC; Burnham and Anderson, 2004), in which a lower AIC is indicative of a better fit.

### Results

#### Confirmatory Factor Analysis

As reported in Table 2, based on the aforementioned fit criteria, the CFAs indicated that Model 4 had not only the best fit among the four hypothesized models, but also was the only one with an adequate fit. Standardized loadings of this model are reported in Table 1. These loadings ranged from 0.55 to 0.86 for the GPF, from −0.10 to 0.54 for the PA factor and, from 0.15 to 0.55 for the PF factor. Items 4, 6, 7, 10, and 13 loaded nonsignificantly on the PA specific factor, indicating that they were almost pure markers of the general broad dimension of prosociality and less markers of PA. Alphas coefficients for PA and PF factors were 0.91 and 0.87 respectively, and 0.94 for the GPF.

**Table 2:** Goodness-of-fit of alternative models for the Prosociality Scale (Study 1).

	*χ*^2^	*df*	CFI	AIC	RMSEA
Model 1	628.913	104	0.858	11760.759	0.119
Model 2	531.174	103	0.884	11665.020	0.108
Model 3	565.564	104	0.875	11697.410	0.111
Model 4	314.702	89	0.939	11476.548	0.081

CFI, Comparative Fit Index; AIC, Akaike information criterion; χ^2^, chi-square statistic; df, degrees of freedom.

#### Construct Validity

Construct validity was assessed by examining correlations between adjustment or maladjustment outcomes and factor scores for the general and specific factors from the bi-factor model. Because observed composite scores for the three factors of the Prosociality Scale do not separate the unique effects of each facet from the shared variance among the facets (GPF, PA, and PF), individuals’ factor scores for GPF, PA and PF were calculated from the bi-factor model loadings (Sinharay and Haberman, 2007) in MPlus. Even though some items did not load significantly in some factors, all of them loaded on at least one factor, therefore factor scores were computed using all the items comprised in Model 4. Table 3 presents the means and standard deviations of variables along with correlations, GPF was significantly and strongly positively correlated with agreeableness, self-transcendence values, and the two dimensions of the empathic responding (i.e., empathic concern and perspective talking). Correlations among variables with the specific factors (PF and PA) were mostly significant, albeit usually lower in size compared to those with the GPF. Correlations of the GPF and PA (but not PF) with the quality of friendship were positive and significant, whereas those with aggression were negative and significant; however, these correlations were small to moderate in size: The correlations generally support the validity of the factors derived from the bifactor model.

**Table 3:** Descriptive statistics and correlations among the factors of the Prosociality Scale and indicators of (Mal) adjustment.

	Prosociality (GPF)	Prosocial actions (PA)	Prosocial feelings (PF)	*Mean*	*SD*
Quality of friendship	0.338^*^	0.3214^*^	0.087	4.161	0.59
Agreeableness	0.595^*^	0.156^*^	0.114^*^	3.412	0.42
Self-transcendence values	0.617^*^	0.109^*^	0.134^*^	4.642	0.56
Empathic concern	0.398^*^ (0.545^*^)	0.278^*^ (0.141^*^)	0.218^*^ (0.153^*^)	0.3.451 (3.523)	0.857 (0.406)
Perspective talking	0.578^*^ (0.610^*^)	0.236^*^ (0.382^*^)	0.281^*^ (0.311^*^)	3.736 (3.695)	0.565 (0.681)
Aggression	−0.285^*^	−0.151^*^	−0.089	1.541	0.448

Data for China are in parentheses.

* *p* < 0.01.

## Study 2

The primary goal of this second study was to assess the cross-cultural invariance of the best model of the Prosociality Scale tested in Study 1 (i.e., bifactorial model). In addition to Italy other four different countries (i.e., China, Chile, Spain, and the United States) were included. These samples were chosen due to a need for a robust test of comparability across dissimilar cultural contexts by considering a non-western-country (i.e., China), a typically western country (i.e., the United States), a Latin American country (i.e., Chile), and a European country other than Italy (i.e., Spain). These nations were also chosen because ongoing collaborations among scholars allowed the collaborative data collection. Note that in bifactor models, measurement invariance of the domain specific factors, in addition to the general factor, can be tested across different groups.

The secondary aim of Study 2 was to further support the construct validity of the Prosociality Scale. Considering the availability of relevant measures, we examined correlations of GPF, PA and PF with empathic concern and perspective talking ability (Davis and Franzoi, 1991) in the Chinese sample as an additional test of convergent validity.

### China, Chile, Italy, Spain, and the United States: Background Information

China, Chile, Italy, Spain, and the United States are countries that differ greatly in terms of language (i.e., Chinese, Italian, Spanish and English), socioeconomic factors, and cultural features. In the Global Competitiveness Index (Central Intelligence Agency, 2016), which measures the economic and political growth of a country, China ranks 28th, whereas Chile is the 33th (the first in Latin America), Italy ranks 44th, Spain ranks 32th, and the United States ranks 3rd. Researchers have noted both differences and similarities among these countries in regard to cultural values related to the enactment of prosocial behaviors (i.e., benevolence, universalism, cooperation, and solidarity; see Vaish and Warneken, 2012; Eisenberg et al., 2015). Whereas benevolence is related to concern for the well-being of people in close relationships (family, school, neighborhood), universalism is related to behaviors associated with helping people and society as a whole; both values are considered representative of a broad self-transcendence value (Schwartz, 2006). Comparisons indicate that people in West European countries, including Italy and Spain, attribute significantly more importance to universalism/benevolence values than in other regions of the world (Schwartz, 2006). Notwithstanding this, it has been underlined that within the Western European region, contrary to other regions, there is a significant heterogeneity among the countries that provide support for comparisons between western European countries. In contrast, the culture in China, as in other Confucian-influenced countries, is mainly concerned with hierarchy rather than with egalitarianism and harmony. Furthermore, although Chinese culture is becoming less traditional than previously, low scores on autonomy/embeddedness suggest that the culture still stresses the importance of reinforcing positive interpersonal ties, albeit mainly to the in-group (Schwartz, 2006). Moreover, different studies have highlighted changes in the social structure and the values of the Chileans, from a more collectivist orientation to, currently, a greater focus on individual development and self-oriented values (e.g., Fernandez et al., 1997). Indeed, findings presented by Schwartz (2006) underlined that although Chile, similarly to other Latin American countries, may be considered a collectivistic culture when compared with Western European countries, this is not the case when comparison is made with, for example, Confucian-influenced cultures. Finally, when Schwartz and Bardi (2001) compared ratings of values in the United States with the pan-cultural norms (of a sample of other 68 countries), benevolence was a little above the average, whereas universalism was extremely low. Further, Schwartz (2006) highlighted the limited emphasis on harmony in the US culture, which at least to some extent accounts for the stereotypical perception of United States as a country promoting egoistic attitude and behavior, even though this is counterbalanced by a focus on religion and traditional family values.

Bearing in mind all these distinctive features, we examined the degree to which Model 4 (the best model in Study 1), with two core dimensions of prosocial tendencies (i.e., actions and feelings) plus a GPF, fit the structure of self-reported prosociality in culturally different contexts.

### Method

#### Participants

Participants were 1.630 young adults coming from China, Chile, Spain and the United States ranging in age from 16 to 35 years (general *M*_age_ = 21,34; *SD* = 3.34). Participants from China numbered 149 ranging in age from 19 to 35 years (30.54% males; 69.46% females; *M*_age_ = 28.37, *SD* = 7.81) and most of them were in college and had a bachelor degree (52.22%), 32.87% had a Master degree or above, and the remaining percentage had a lower educational level (i.e., high school or less). The Chilean sample was composed by 451 college students ranging in age from 19 to 33 years (46.31% males; 53.68% females; *M*_age_ = 21.19; *SD* = 2.27) drawn from the urban area of Santiago de Chile. The sample in Spain included 116 college students ranging in age from 19 to 35 years (30.2% males; 69.8% females; *M*_age_ = 26.84; *SD* = 4.13). The U.S. participants were 914 college undergraduate students ranging in age from 19 to 22 years old (51.7% males, 49.3 females; *M*_age_ = 19.55 years; *SD* = 0.86). The majority of the US participants were Caucasian (70.9%), 10% were Hispanic, 5% were Asian, 2.9% were African American, 1.4% were Middle-Eastern, and the remaining participants declared none (1.4%) or two (5.3%) or three (0.5%) of the previous ethnicities.

#### Procedures

Date collection differed across the five samples, although there was an effort to obtain samples somewhat similar in socioeconomic status. In China, all participants were tested *via* an online survey. The survey was posted on a professional website which is specifically and widely used for surveys in China. Participants were also invited by a widely used Chinese social network on the web. Participants were randomly picked to receive a small gift from the website after the survey.

In the Chilean and the US samples, researcher assistants administered questionnaires that included the Prosociality Scale during class. Participation was voluntary, although it was one of many activities that students could select to fulfill a requirement for their introductory psychology class (*blinded for review*). In Spain, participants were recruited from university courses (*blinded for review*) and answered questionnaires individually at their homes. These college students received credit points for their participation. The Italian sample was presented in Study 1.

A procedure of forward- and back-translation was used to ensure the linguistic and conceptual equivalence of measures across languages (see Maxwell, 1996). Measures created in English were translated and administered in Mandarin Chinese (China), Spanish (Chile and Spain), and Italian (Italy). Two different translators, fluent in the original language scales and the target languages, did the first translation and the linguistic adaptation of items. The back translation was performed by another different translators. When doubts regarding the meaning of items emerged, translators discussed and arrived at a proposal that, in turn, was exposed to 4–5 different young individuals (with similar sociodemographic characteristics of participants of the study) and from their understanding the more accurate language was adopted.

#### Measures

##### Prosociality Scale

The same prosocial measure from Study 1 was used in this study. Items were presented in the same order in all samples.

#### Analytical Approach

The bifactor model was first tested separately by country and the goodness fit of these models was evaluated following the same criteria as in Study 1. The same program from Study 1 (Mplus 7.11; Muthén and Muthén, 2012) was used for these analyses. Then we examined the measurement invariance of the bifactor model across countries by using a multigroup confirmatory factor analysis. The equivalence between the four groups was evaluated by including constraints imposing identical unstandardized estimates for the model’s parameters across countries. In particular, three levels of invariance were tested: configural invariance (i.e., same factor structure across groups for the measure of prosocial behavior), metric invariance (i.e., same pattern of factor loadings across groups), and scalar invariance (i.e., the same intercepts of items’ regressions on the latent variables across groups). The plausibility of equality constraints among groups is usually examined with the *χ*^2^ difference test between nested models (i.e., constrained vs. the baseline unconstrained models), in which the invariance is supported if the equality constraints produce a nonsignificant increase of the chi-square. However, following suggestions made by Cheung and Rensvold (2002), as well as other recent studies (e.g., Alessandri et al., 2015), and knowing that this test has substantial power in large samples (Kline, 2005), we also examined the ∆CFI index. Scholars consider a difference in CFI larger than 0.01 as indicative of a meaningful change in model fit (Cheung and Rensvold, 2002). Although we present both ∆*χ*^2^ and ∆CFI, we based our decisions regarding invariance among countries on the ∆CFI index.

### Results

The bifactor model had an adequate fit within each country (see Table 4). Factor loadings are presented in Table 5. All items loaded significantly in all four countries on the GPF. In regard to the factor loadings on specific factors, however, there were some differences. In China, items 6, 7, 9, 10, 13, and 15, loaded nonsignificantly on the PA specific factor, whereas item 5 loaded nonsignificantly on the PF specific factor. In Chile, items 10, 11, and 14 loaded nonsignificantly on the PA specific factor. In Spain, items 3, 4, 6, 7, 10, 11, and 13 loaded nonsignificantly on the PA specific factor, whereas item 16 loaded nonsignificantly on the PF specific factor. Finally, in the United States, items 10, 13, and 15 loaded nonsignificantly on the PA specific factor, and item 5 loaded nonsignificantly on the PF specific factor. These nonsignificant factor loadings on specific factors suggest that those items were pure markers of the broad tendency to respond prosocially. Alphas coefficients for the GPF were 0.91 in China; 0.87 in Chile; 0.92 in Spain; 0.94 in Italy; and 0.90 in the US Alphas for PA and PF factors were, respectively, 0.90 and 0.61 in China; 0.83 and 0.70 in Chile; 0.83 and 0.72 in the United States; 0.91 and 0.87 in Italy; and 0.87 and 0.76 in Spain.

**Table 4:** Summary of fit statistics for cross-cultural invariance of the best-fitting model (Study 2).

	*χ*^2^	*df*	CFI	∆*χ*^2^	∆*df*	∆CFI
China	150.748^*^	89	0.946	–	–	–
Chile	300.399^*^	89	0.903	–	–	–
Spain	173.052^*^	89	0.905	–	–	–
United States	590.282^*^	89	0.906	–	–	–
Italy	314.702^*^	89	0.939	–	–	–
Configural	1560.603^*^	449	0.917	–	–	–
Metric	2071.786^*^	569	0.887	511.183^*^	120	−0.029
Metric_Partial_	1803.020^*^	556	0.906	242.417^*^	107	−0.010
Scalar	2700.89^*^	619	0.843	897.87^*^	63	−0.063
Scalar_Partial_	1920.928^*^	599	0.901	117.908^*^	43	−0.006

CFI, Comparative Fit Index; TLI, Tucker Lewis Index; RMSEA, root mean square error of approximation; AIC, Akaike information criterion; χ^2^, chi-square statistic; df, degrees of freedom.

* *p* ≤ 0.05.

**Table 5:** Standardized factor loadings in the bifactorial model from CFA of the Prosociality Scale separately for the countries.

Items	China		Chile		Spain		US	
	GPF	SF	GPF	SF	GPF	SF	GPF	SF
PA								
Item 1	0.469^*^	0.595^*^	0.443^*^	0.625^*^	0.499^*^	0.209^*^	0.432^*^	0.513^*^
Item 2	0.518^*^	0.591^*^	0.421^*^	0.200^*^	0.599^*^	0.559^*^	0.404^*^	0.537^*^
Item 3	0.682^*^	0.537^*^	0.489^*^	0.592^*^	0.728^*^	−0.019	0.567^*^	0.386^*^
Item 4	0.617^*^	0.334^*^	0.436^*^	0.316^*^	0.569^*^	−0.189	0.494^*^	0.169^*^
Item 6	0.746^*^	0.117	0.637^*^	0.217^*^	0.815^*^	0.074	0.585^*^	0.117^*^
Item 7	0.771^*^	0.182	0.623^*^	0.316^*^	0.733^*^	−0.125	0.545^*^	0.101
Item 9	0.665^*^	0.009	0.438^*^	0.253^*^	0.658^*^	0.177^*^	0.563^*^	0.251^*^
Item 10	0.562^*^	−0.040	0.668^*^	−0.100	0.735^*^	0.022	0.717^*^	0.020
Item 11	0.628^*^	0.188^*^	0.443^*^	−0.028	0.526^*^	0.061	0.476^*^	0.219^*^
Item 13	0.773^*^	−0.057	0.724^*^	0.161^*^	0.748^*^	−0.010	0.751^*^	0.011
Item 14	0.630^*^	0.226^*^	0.428^*^	0.014	0.628^*^	0.399^*^	0.580^*^	0.334^*^
Item 15	0.653^*^	−0.161	0.600^*^	0.056	0.677^*^	0.200^*^	0.649^*^	0.079
PF								
Item 5	0.520^*^	0.176^*^	0.614^*^	0.510^*^	0.604^*^	0.391^*^	0.635^*^	−0.040
Item 8	0.499^*^	0.662^*^	0.628^*^	0.414^*^	0.695^*^	0.416^*^	0.628^*^	0.278^*^
Item 12	0.576^*^	0.529^*^	0.356^*^	0.157^*^	0.498^*^	0.448^**^	0.685^*^	0.451^*^
Item 16	0.518^*^	0.531^*^	0.300^*^	0.186^*^	0.603^*^	−0.121	0.525^*^	0.017^*^

SF, Specific Factors; PA, Prosocial Actions; PF, Prosocial Feelings; GPF, General Prosocial Factor.

Data for Italy is reported in Table 1.

* *p* ≤ 0.05.

### Cross-Cultural Measurement Invariance

As reported in Table 4, the configural and metric invariance model fit the data well. Nevertheless, the addition of equality constraints among countries on item loadings (i.e., comparing the configural with the metric invariance model) worsened the model fit (see Table 4). Modification indices were used to refine the structural models (Steenkamp and Baumgartner, 1998) and partial metric invariance was reached after releasing the equality constraints of the factor loadings for (1) item 5 on PA in the five countries; (2) item 16 on PF in China; (3) items 2, 11, and 12, 14 on GPF in Chile; (4) item 15 on PA in Italy; (5) item 13 on PA in the US; and (6) the residual variances for the latent factor for PA in Chile, PF in China, and GPF in the United States. According to the ∆CFI index (see Table 4), this weaker metric-invariant model fit the data well and was not significantly different from the configural model.

The scalar invariance model did not fit the data well and its fit was significantly worse than the previous partial metric model (see Table 4). We followed the same prior procedure and test the plausibility for a partial scalar invariance by relaxing constraints imposed on the intercepts of (1) items 4, 7, 15 and 16in China; (2) items 5, 7, 11, 12, 13, and 14 in Chile; (3) items 7 and 15 in Spain; (4) items 6 and 8 in US; (5) items 1, 5, 7, and 8 in Italy. In summary, these results support the conclusion that the hypothesized model is partially invariant across five different countries. Configural, partial metric, and partial scalar invariance are indicators of the robustness of the Prosociality Scale.

### Construct Validity

To provide further evidence of the construct validity of the Prosociality Scale, following the same procedure as in Study 1 and using available measures, we correlated the empathic concern and perspective taking subscales (*α*_s_ = 0.79 and 0.84, respectively) with the factors of the Prosociality Scale in the Chinese sample. Both empathy subscales were weakly to moderately, positively related to PA and PF, and moderately to strongly related to GPF (see Table 3).

## Discussion

It is only in recent decades that researchers and practitioners have begun to pay attention to the development of skills and competencies that support better societal conditions and individuals’ positive well-being such as prosocial behaviors (Greenberg et al., 2001). The current investigation sought to provide evidence of the psychometric properties and the cross-cultural generalizability of a measure to assess the tendency to enact prosocial behaviors. In particular, this study can be considered a step forward in the measurement of a global tendency to behave in prosocial ways during late adolescence and early adulthood. It builds on previous studies on the psychometric properties of the Prosociality Scale (Caprara et al., 2005a,b) by assessing the multidimensional factorial structure of this scale (in Italy) and its generalization to five other Western and non-Western countries (China, Chile, Spain, and the United States).

First, in Study 1, we compared 4 different structural solutions for the Prosociality Scale and findings supported the existence of a bifactor model in which there are two specific latent factors, the PA and PF factors, plus a general (unrelated) latent factor, likely associated with a systematic tendency to behave in favor of others. The bifactor model has been used to examine constructs that are comprised of multiple related yet distinct facets (Chen et al., 2006; Reise et al., 2007). The bifactor model in this study suggested that: (1) there is a general factor that accounts for the commonality of prosocial tendencies shared by the facets that are different ways to react when people are probed to behave prosocially (*via* specific actions or specific feelings), and (2) there are two main specific factors, each of which accounts for the unique influence of the specific facet over and above the general disposition to enact prosocial behaviors. These findings indicate that the scale includes elements relevant to both the general tendency to be prosocial, as well special types of prosocial behaviors, although researchers using the scale have not previously sorted out the variance related to general vs. specific components. Indeed, in line with prior suggestions (Caprara et al., 2005b), these results highlight that prosocial actions and feelings assess two aspects of prosocial tendencies, both different from a general disposition, at least starting at the end of adolescence. The GPF may also reflect what is common to both but different from what affects the tendency to respond specifically with action vs. feelings.

Moreover, in Study 2, multigroup CFAs allowed for corroboration of the bifactorial structure of the scale, demonstrating configural invariance and both partial metric and partial scalar invariance in four representative countries of western and non-western cultures. General and specific factors were found in all four samples. However, these findings have to be considered with some caution because in the comparison of models for the five countries, metric invariance held for most items, robustly supporting the strength of the factorial structure of the scale in the five countries. Scalar invariance was less strongly supported, although some items were comparable across some groups. By analyzing scalar invariance, it is possible to identify a subset of invariant items for each factor (PA and PF). This means that the scale can also be considered a good instrument to assess the tendency to enact prosocial behaviors among older youths and adults and that a subset of items may also allow for robust mean comparisons across national groups.

The current findings can be considered important for several reasons. First of all, to our knowledge, this is the first cross-cultural validation of a measure for testing global prosocial tendencies in young adult populations in quite different countries. Furthermore, evidence of construct validity was found for the two countries for which data were available (Italy and China). Italy and China may be considered representative of very dissimilar cultural contexts, supporting our conclusion regarding the validity of the Prosociality Scale. Prosociality, as expected, was correlated with both general indicators of adjustment (positively) and maladjustment (negatively).

These results indicate that at a general level this scale is able to measure a tendency to act prosocially in various countries and therefore indicates a relatively universal pattern of measuring prosocial behavior. Moreover, it is expected that this scale will identify cultural differences in the tendencies to act and feel prosocially. Indeed, it also is important to recognize the role of cultural values in investigating national differences in prosocial responding. The participating countries varied widely not only on socio-demographic indicators, but also on psychological constructs such as individualism vs. collectivism. Using Hofstede’s (2001) rankings, the participating countries ranged from the United States, with the highest individualism score in the world to China, countries that are among the least individualistic in the world. We hope that the Prosociality Scale will allow us to capture cultural differences in levels of adherence to prosocial behavior and its manifestations in actions and feelings.

The potential applicability and utility of the Prosociality Scale is broad. Because prosociality has been found to be highly correlated with well-established indicators of well-being and adjustment, researchers in the areas of Positive Psychology (Seligman and Csikszentmihalyi, 2000) and Positive Youth Development (Lerner et al., 2005) might benefit from using this broad, brief, reliable, and easily administered instrument to assess individual tendencies in the enactment of prosocial behavior across situations. Because the tendency to act in favor of others has been also identified as playing a significant role in contrasting a variety of psychological dysfunctions, such as depression and externalizing behaviors (see Penner et al., 2005), this scale may also have utility for clinical purposes and preventive efforts. Indeed, because the use of this scale is less costly than other experimental measures of prosocial behavior, it can be also used in large intervention programs. Likewise, because the study of the development of prosocial behaviors across adulthood is still scarce, the scale could be useful for examining specific mechanisms involved in prosocial development across this developmental phase.

Future research should address and overcome several potential limitations in the current study. First, a multi-method approach could highlight the extent to which self-reported behaviors are consistent with others’ reports in different cultures (or other indices of prosociality). In addition, because it is reasonable to expect that people overestimate their own prosocial behavior, it would be useful to include a measure of, and control for, social desirability. In general, prosocial behaviors, because they benefit others, tend to be desirable, so individuals may be more likely to report helping experiences that are considered to be socially acceptable. Because our samples were not representative, caution on using and interpreting these results should be taken. Regarding findings of some nonsignificant factor loadings on specific factors in some sample countries, it is unlikely that the value of any specific loading is completely equal to zero and that the values are, to some extent, due to some specific characteristic of our sample. Therefore, we recommend that scholars check the values of the nonsignificant factor loadings on specific factors to see if they vary across samples. Moreover, future studies should consider testing a shortened version of the measure in order to obtain a more balanced solution of the two factors and a more cost-effective and widely applicable version of the scale.

Despite all these limitations, we believe that this study represents a step forward in the assessment of prosocial behaviors in different cultural contexts by fostering adequate cross-national comparisons.

## Data Availability Statement

The raw data supporting the conclusions of this article will be made available by the authors, without undue reservation.

## Ethics Statement

The studies involving human participants were reviewed and approved by Sapienza University of Rome. The patients/participants provided their written informed consent to participate in this study.

## Author Contributions

BL conceived the study, participated in its design, performed the statistical analysis, and coordinated and drafted the manuscript. NE participated in the design of the study and drafted and corrected the manuscript. CT participated in the design of the study, coordinated data collection in Chile, and drafted the manuscript. AZ collaborated in the revision of statistical analyses and its interpretation and drafted the manuscript. MC coordinated data collection in Spain and drafted and corrected the manuscript. ER coordinated data collection in Chile and drafted and corrected the manuscript. LZ coordinated data collection in China and drafted and corrected the manuscript. CP and GC participated in the design of the study, coordinated data collection in Italy, and drafted and corrected the manuscript. All authors read and approved the final manuscript.

### Conflict of Interest

The authors declare that the research was conducted in the absence of any commercial or financial relationships that could be construed as a potential conflict of interest.

## Footnotes

^1^Because a single second-order factor with two first-order factors results in a non-identified model, to enable identification, we imposed equality constraints to the factors loadings of the two first-order factors on the second-order factor, as well as on residual variances of the first-order factors.
